# Timescale Halo: Average-Speed Targets Elicit More Positive and Less Negative Attributions than Slow or Fast Targets

**DOI:** 10.1371/journal.pone.0083320

**Published:** 2014-01-08

**Authors:** Ivan Hernandez, Jesse Lee Preston, Justin Hepler

**Affiliations:** Department of Psychology, University of Illinois at Urbana-Champaign, Champaign, Illinois, United States of America; University of Bath, United Kingdom

## Abstract

Research on the *timescale bias* has found that observers perceive more capacity for mind in targets moving at an average speed, relative to slow or fast moving targets. The present research revisited the timescale bias as a type of halo effect, where normal-speed people elicit positive evaluations and abnormal-speed (slow and fast) people elicit negative evaluations. In two studies, participants viewed videos of people walking at a slow, average, or fast speed. We find evidence for a *timescale halo effect*: people walking at an average-speed were attributed more positive mental traits, but fewer negative mental traits, relative to slow or fast moving people. These effects held across both cognitive and emotional dimensions of mind and were mediated by overall positive/negative ratings of the person. These results suggest that, rather than eliciting greater perceptions of general mind, the timescale bias may reflect a generalized positivity toward average speed people relative to slow or fast moving people.

## Introduction

A growing body of work has pointed to the importance of dynamic motion cues in making inferences about others' minds [1]. For example, people readily make judgments about emotions of targets in point-light displays, where movement patterns are observable even though all other physical cues are absent [2], [3]. Observers also use the direction of movement to make spontaneous judgments about the intentions and goals of an agent [4]. The relative speed of an actor serves as another important kind of movement cue. For example, studies on the *timescale bias* [5] show that targets moving at an average human speed are perceived by observers as having more capacity for “mind” than targets moving at either a slow or fast pace. Researchers explained the pattern as an anthropocentric bias in anthropomorphism—that is, those targets that appeared most like a typical human were attributed more human-like characteristics. The explanation that something having human-like speed is more likely to be perceived as being human-like has an intuitive appeal as well. This interpretation of the timescale bias was consistent with the emerging literature on agency detection [6]–[8], and subsequent research in mind perception and anthropomorphism [9]–[11].

### Timescale Halo

In the present research, we examine whether the timescale bias in human targets may emerge as a *halo effect*, such that human targets walking at an average speed are judged more positively, in general. In other forms of the halo effect, a person who is judged positively on one trait is also rated positively on other, often, unrelated specific traits, and this effect is mediated by general feelings of liking [12], [13]. For example, physically attractive targets receive more favorable personality assessments [14], writing evaluations [15], and court sentences [16] than their less attractive counterparts receive. Similarly, for the timescale bias, the relative speed of a target may affect global evaluations such that people who are walking at normal, average speeds are rated more positively than slow or fast moving targets. This positivity could then generalize to a variety of unrelated mental traits. We speculate such a *timescale halo effect* may occur for two reasons. First, those walking at an average speed may seem more like oneself, whereas people moving at relatively slow or fast speeds may appear very dissimilar to the self. Generally, people like similar others more than dissimilar others [17], [18] and are better able to imagine their point of view, which can enhance empathy [19]. Second, actors who move especially slow or fast may appear strange or abnormal, and this abnormality can prompt negative judgments [20], [21]. Characteristics that are extreme on some dimension (such as extremely fast/slow movement) attract greater attention from observers, and subsequently carry more weight when forming a final impression of others [22]. Negative attributions are especially likely to influence impression formation because they are weighed more strongly than positive attributes by the observer [23], [24]. Thus, people moving at slow or fast speeds may appear abnormal or very different from oneself, which can affect the overall liking for the target. As a result, people who move at an average or typical speed may be attributed with more positive attributes and fewer negative attributes than slow or fast people, including their mental capacity.

The present research examines this account of the timescale bias using a broad array of mental traits. The original timescale bias studies treated “mind” as a general capacity that may have been considered a positive attribute (it is certainly better to have more “mind” than less “mind”). However, having a mind is best described as a broad capacity to engage in a variety of more specific mental experiences (e.g., the ability to contemplate, imagine, plan, decide, analyze, compute, feel). There are many specific attributes of mind that are strongly negative (e.g., deceit, manipulation, scheming) that were not included in the composite measures of the original timescale studies. The original timescale bias research focused on the relative quantity of a mind (“Is the target smart?”), but do not speak to other qualitative aspects of mind (“Is he scheming?”,“Is he creative?”) that imbue it with positive or negative characteristics. This differentiation between positive and negative mental traits provides a unique means to distinguish between two alternative explanations of the timescale bias. If the timescale bias pattern is the result of an anthropocentric bias in mind perception, average-speed targets should be perceived to have a greater capacity for all kinds of mental characteristics—whether they are positive (e.g. more intelligent) or negative (e.g., more deceitful). On the other hand, if the timescale bias pattern is the result of a *timescale halo effect*, average-speed targets should be attributed with more positive traits (e.g., more intelligent) than slow or fast targets, but also attributed with *fewer* negative traits (e.g., less deceitful) than slow or fast moving targets, even if those trait signify greater mental capacity.

In addition to a positive or negative valence, it is worth noting that mental characteristics also vary on cognitive and emotional dimensions. Gray, Gray, and Wegner [25] distinguish between mental capacities of *agency* (e.g. consciousness, intentionality, analysis) and *experience* (e.g. emotion, sensation), and research on person perception has revealed that judgments of others vary on *warmth* and *competence* [26], [27]. The warmth dimension reflects interpersonal or social characteristics (e.g., honest, tolerant), whereas the competence dimension reflects whether a target is skillful and can successfully act on intentions and goals (e.g., determined, imaginative). These models each contrast cognitive and emotional components of mind, but are independent from the positive/negative valence of the trait. For example, the capacity to be *rational* and the capacity to be *deceptive* could both be highly cognitive traits, requiring a great deal of thought and agency. Whereas rationality is considered a desirable trait in others, deceptiveness is highly undesirable. Likewise, the capacity for *joy* and the capacity for *irritability* both reflect emotional traits, but joy is a far more positive characteristic than irritability. The original timescale bias research only studied cognitive components of mind (i.e. intention, intelligence), so it is important in the present research to extend these analyses to emotional components as a more comprehensive test of speed and mental attributions. If movement speed influences observers' judgments of a target in a timescale halo, we should expect a similar halo pattern for both cognitive and emotional dimensions of mind.

### The Present Research

The present research examines a *timescale halo hypothesis*, i.e., that the speed of a person affects attributions of mind because of the general positivity felt towards the target. In two studies, participants observed human targets walking at either an average, slow, or fast speed relative to other pedestrians. They then rated each target on several attributes that included positive, negative, cognitive and emotional traits. People who move at a typical, average human speed should garner more favorable impressions that then generalize to a variety of specific positive emotional and cognitive traits. On the other hand, people who move slower or faster than average should seem to have more negative emotional and cognitive traits. Moreover, we predict that this pattern should be mediated by global feelings of positivity/negativity towards the targets. Such results would suggest that moving at an average speed does not enhance overall attributions of mind, but rather enhances attributions of positive characteristics over negative characteristics.

## Pilot Study

As an initial examination of how speed affects mental trait ratings in general, we conducted a pilot study examining the timescale bias using a variety of specific mental traits (e.g., *conscious, deceptive, sentimental, irritable*). In this study, we examined whether the timescale bias' prediction (i.e. that targets walking at average human speeds should be attributed more mental capacity) would hold for a broader selection of mental traits than originally examined. The results could offer insight into whether the timescale bias' findings apply to all positive/negative traits for different dimensions of mind, or if the average-speed effect is restricted to only a subset of traits. We followed the procedures described by Morewedge et al. [5] by having participants watch three separate videos that each featured a target person walking on a busy sidewalk. Relative to other pedestrians in the video, the target's pace was either slow, average, or fast. After each video, participants rated each target on a list of specific traits that included many different cognitive and emotional attributes.

### Method

#### Ethics statement

This research protocol was reviewed and approved by the Institutional Review Boards for Research with Human Participants at the University of Illinois at Urbana-Champaign. Participants provided written informed consent prior to engaging in research activities. This research was conducted in accordance with the standards set forth by the American Psychological Association.

#### Participants

51 undergraduates from University of Illinois participated for partial course credit.

#### Stimuli

In a repeated-measures design, participants watched three separate videos (courtesy of Carey Morewedge [5]) that each featured a different target person walking on a busy sidewalk. Before each video began, a single frame from the video showed the target circled in red, and participants were instructed to watch that person during the video. Targets moved at either an average pace relative to other pedestrians (*M* = 3.20 mph), a slower-than-average speed (*M* = 1.03 mph), or faster than average speed (*M* = 4.99 mph). Three different versions of the videos were produced that counterbalanced target person with walking speed, and these versions were randomly assigned to participants. Participants never rated the same target more than once and never saw the same walking speed more than once. Videos were presented in random order.

#### Mental Attributions

The list of mental traits was generated in pretesting and selected to represent both cognitive and emotional aspects of mind, and both positive and negative attributes. Forty-one pretest subjects rated 200 trait words on “How much thinking is required to have this characteristic?” and “How much feeling is required to have this characteristic?” using scales from 1 (*requires very little thinking/feeling*) to 7 (*requires a lot of thinking/feeling*). Positive/negative valence for each trait was assessed by the question “How good do you think the trait is to have?” using a scale of 1 (*negative*) to 7 (*positive*). We created a cognitive trait list and an emotional trait list by selecting the five items that were rated as requiring the most “thinking” (*attention*, *conscious*, *deceptive*, *planning*, and *rational*), and the most “feeling” (*feels pain*, *feels pleasure*, *irritable*, *passionate*, *sentimental*, and *warmth*), ensuring that each list include at least one undesirable trait (*deceptive, feels pain, irritable*).

In the current study, participants rated each target on their capacity for each of these 11 mental traits, immediately after each video. Ratings were made on a 7-point scales (e.g., “How capable is the target of being rational?” from 1 = *“Not at all”* to 7 = *“Extremely”*).

### Results

#### Positive vs. Negative attributions

**Figure 1:**
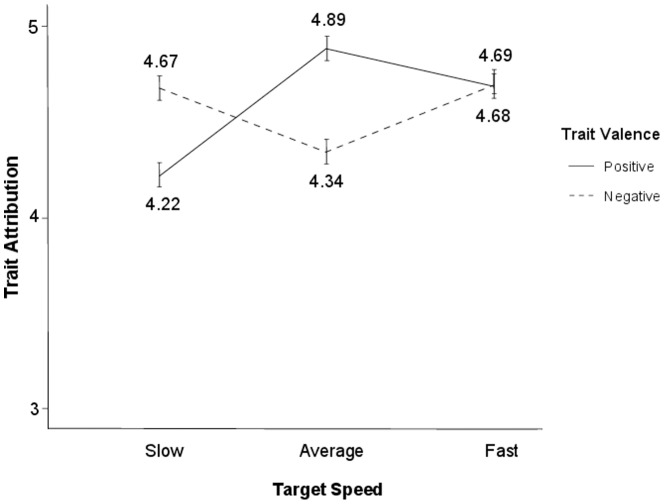
Attribution of targets' capacity for positive traits and negative traits, Pilot Study. Ratings were made from 1 (Not at all) to 7 (Extremely).

Our first analysis examined whether average-speed targets were more likely to receive positive than negative attributions of mind, which would be consistent with the timescale halo prediction. We computed the reliabilities for the positive and negative items for each separate speed and then averaged those reliabilities into a composite index of reliability. The average Cronbach's alpha of the positive traits showed high reliability, α_Positive_ = .78 (α_slow_ = .76, α_average_ = .83, α_fast_ = .74). The negative traits, however, demonstrated relatively low reliability, α_Negative_ = .46 (α_slow_ = .41, α_average_ = .64, α_fast_ = .35). To further investigate whether attributions of mind differ for positive and negative traits in general, we collapsed across those items to form a positive composite and a negative composite scale. Averaging across the traits creates a general measure of the traits' construct, which gives equal weighting to all traits included and does not assume one trait contributes more to the construct than any other does. Although other weighting approaches can be used, unit weighting is associated with less sampling error and is less susceptible to outliers [28], [29]. This approach was also used by Morewedge et al. in their original studies, allowing our results to be easily compared with theirs. The means of the positive and negative attributions were analyzed by a 2 (Valence: Positive, Negative)×3 (Speed: Slow, Average, Fast) repeated-measures analysis of variance (ANOVA). There was no main effect of valence (*F*<1), but there was a main effect of speed, *F*(2, 50) = 3.85, *p*<.05, η^2^_partial_ = .07. Importantly, these effects were qualified by the predicted interaction between valence and speed, *F*(2, 50) = 15.88, *p*<.001, η^2^_partial_ = .24 (see Figure 1). To explore this interaction, we conducted simple effects tests. As predicted, the average-speed target received stronger attributions on positive traits (*M* = 4.89, *SD* = .70) than slow (*M* = 4.22, *SD* = .85, *p*<.001) and fast targets (*M* = 4.68, *SD* = .80, *p*<.05; see Table 1 for all means). This pattern replicated the effect observed in the original timescale bias paper. However, the opposite pattern was observed for negative traits—consistent with the timescale halo hypothesis—average-speed target received weaker attributions for negative traits (*M* = 4.34, *SD* = .92) than slow (*M* = 4.67, *SD* = .91, *p*<.05) and fast targets (*M* = 4.69, *SD* = .85, *p*<.01).

**Table 1:** Perceived capacity for traits by target speed, Pilot Study.

			Target Speed					
			Slow		Average		Fast	
Trait	Valence	Dimension	Mean	(SD)	Mean	(SD)	Mean	(SD)
*Attention*	Positive	Cognitive	4.18	(1.72)	5.04	(1.10)	4.80	(1.48)
*Conscious*	Positive	Cognitive	5.16	(1.41)	5.24	(0.94)	5.22	(1.27)
*Planning*	Positive	Cognitive	4.12	(1.28)	4.98	(0.88)	4.59	(1.51)
*Rational*	Positive	Cognitive	4.45	(1.21)	4.96	(0.92)	4.80	(1.23)
*Passionate*	Positive	Emotional	3.84	(1.39)	4.69	(4.69)	4.92	(1.26)
*Sentimental*	Positive	Emotional	4.18	(1.47)	4.53	(1.35)	4.53	(1.10)
*Warmth*	Positive	Emotional	3.78	(1.00)	4.73	(1.28)	4.20	(1.47)
*Feels Pleasure*	Positive	Emotional	4.04	(1.28)	4.94	(1.19)	4.41	(1.27)
*Deceptive*	Negative	Cognitive	4.18	(1.28)	4.24	(1.14)	4.33	(1.29)
*Feels Pain*	Negative	Emotional	5.06	(1.49)	4.86	(1.17)	4.82	(1.37)
*Irritable*	Negative	Emotional	4.78	(1.25)	3.92	(1.29)	4.04	(1.21)

*Means and standard deviations of the ratings given for each target assessing their capacity for various mental traits at different walking speeds.*

#### Cognitive vs. Emotional Attributions

We were also interested in whether similar patterns would be observed across both cognitive and emotional traits, independent of valence. Negative traits were reverse-scored so that larger values reflected more positive attributions, and separate means for cognitive and emotional trait attributions were calculated for each target (slow, average, fast). We calculated the average reliability of the items that went into cognitive and emotional scales, α_Cognitive_ = .43(α_slow_ = .33, α_average_ = .47, α_fast_ = .47), α_Emotional_ = .39(α_slow_ = .51, α_average_ = .29, α_fast_ = .37). These reliabilities were low, which may indicate that traits do not correlate together well as separate cognitive vs. emotional categories. A 2 (Trait Category: Cognitive, Emotional)×3 (Speed: Slow, Average, Fast) repeated-measures ANOVA revealed a main effect for trait category, *F*(1, 50) = 50.09, *p*<.01, η^2^_partial_ = .54, where targets were rated more highly on cognitive traits (*M* = 4.59, *SD* = .49) than emotional traits (*M* = 4.02, *SD* = .45). For these analyses, we had reverse-scored negative traits to reflect more positive judgments, so the timescale hypothesis would predict a quadratic pattern across speed. There was indeed a significant main effect for speed, *F*(2, 50) = 20.43, *p*<.001, η^2^_partial_ = .29. Follow-up testing found that the effect is quadratic: *F*(1, 50) = 26.56, *p*<.01, η^2^_partial_ = .32. Average-speed targets received stronger attributions (*M* = 4.57, *SD* = .45) than slow (*M* = 4.01, *SD* = .58) and fast targets (*M* = 4.33, *SD* = .56). The interaction between trait and speed was not significant, *F*(2, 50) = 1.25, *p* = .29, η^2^_partial_ = .02. Additionally, separate quadratic contrasts on the cognitive and emotional traits revealed that the curvilinear trend held for both dimensions. For the cognitive traits, average-speed targets received more positive ratings (*M* = 4.80, *SD* = .57) than slow (*M* = 4.35, *SD* = .72) or fast targets (*M* = 4.62, *SD* = .77; *F*(1, 50) = 7.57, *p*<.01, η^2^_partial_ = .13. Likewise, for emotional traits, average-speed targets were attributed more positive ratings (*M* = 4.35, *SD* = .54) than slow (*M* = 3.67, *SD* = .73) or fast targets (*M* = 4.05, *SD* = .63), *F*(1, 50) = 22.72, *p*<.001, η^2^partial = .31.

### Discussion

Target people moving at an average-speed relative to other pedestrians elicited stronger positive mental attributions than slow or fast moving people. However, we also observed that average-speed targets elicited weaker attributions on negative mental attributions, compared to slow or fast targets. This pattern occurred for both cognitive and emotional characteristics. These results suggest that movement speed interacts with trait attributions as a function of overall positivity, (i.e., a halo effect), rather than greater overall mind attributed to average-speed targets.

These results suggest that there may indeed be a timescale halo effect, but the present study has some limitations. First, our scales included more positive traits than negative traits, and these negative items reflect multiple dimensions, which may have contributed to the low scale reliabilities. Our central predictions depend on a reliable contrast between positive vs. negative mental attributions, so it is critical that we replicate this design with several negative mental attributes for each dimension. Second, we inferred a timescale halo effect based on divergent attributions for negative and positive traits, but we did not ask participants to rate the overall positivity/negativity of the targets in this study. To provide direct evidence for the existence of this halo effect it is important to demonstrate the effect is mediated by general feelings of liking. We addressed both limitations in our next study.

## Main Study

We replicated the basic design of the pilot study with some critical changes. First, we created a new list of mental attributes with equal numbers of positive/negative and cognitive/emotional traits. Additionally, participants rated their general positivity towards each target, and their subjective perceptions of the relative speed of each target. These ratings allowed us to test the timescale halo hypothesis by examining whether global positivity mediated the relation between subjective speed and trait attributions.

### Method

#### Ethics statement

This research protocol was reviewed and approved by the Institutional Review Boards for Research with Human Participants at the University of Illinois at Urbana-Champaign. Participants provided written informed consent prior to engaging in research activities. This research was conducted in accordance with the standards set forth by the American Psychological Association.

#### Participants

Ninety-six undergraduate students at the University of Illinois participated for partial course credit.

#### Video Stimuli

Video stimuli and manipulation were identical to the pilot study.

#### Mental Attribution Scales

We created four separate trait scales using Anderson's 555 personality-trait ratings inventory, which contains both cognitive and emotional traits that have been measured for trait desirability [30]. We sorted scale items by their desirability rating, and then searched the top and bottom 25% of items for traits that were highly cognitive and highly emotional. We created four separate scales with four items each. The positive-cognitive scale (PC) consisted of *clever*, *creative*, *imaginative*, and *smart*. The negative-cognitive scale (NC) consisted of *critical*, *deceptive*, *fault-finding*, and *scheming*. The positive-emotional scale (PE) consisted of *compassionate*, *helpful*, *loyal*, and *patient*. The negative-emotional scale (NE) consisted of *irritable*, *jealous*, *lonely*, and *nervous*.

Participants were asked to rate the capacity of the target on each of the traits using a 7-point scale, for example: “In general, how compassionate is the target?” with endpoints, 1 = *“Not at all”*, 7 = *“Extremely”*.

#### Procedure

Participants were seated at individual computers and given instructions for the task on the screen. They were told that they would be making ratings about various people in a series of videos. Before each video began, a single frame from the video showed the target circled in red, and participants were instructed to watch that person during the video. After viewing each video, participants rated each target (“How positive/negative do you feel towards the target?” from 1 = *extremely negative* to7 = *extremely positive*),completed the PC, NC, PE, and NE scales for each target, and then rated each target based on how each target appeared to move on a scale from 1 (*extremely slow*) to 4 (*normal*) to 7 (*extremely fast*). This rating task was repeated two more times with a different target and speed for each video, so that all participants viewed a target walking at slow, average, and fast speeds. Videos were presented in a random order, such that each participant never rated the same target more than once and never saw the same walking speed more than once.

### Results

#### Manipulation check

We analyzed participants' subjective ratings of speed for each video (slow, average, fast) using a repeated measures ANOVA. There was a significant main effect of target's speed on the perceived target speed, *F*(1, 95) = 754.52, *p*<.001, η^2^_partial_ = .89. Simple effects tests confirmed that slow targets were perceived as slower (*M* = 1.35, *SD* = .78) than average-speed targets (*M* = 3.93, *SD* = .62), and both were perceived as slower than fast targets (*M* = 5.76, *SD* = .96), all *p*s<.001. Furthermore, a one-sample *t*-test revealed that the perceived speed of average-speed targets was not significantly different from the scale midpoint of 4 (labeled *normal* on the scale), *t*(95) = −1.15, *p* = .25, indicating that participants perceived average-speed targets as normative.

#### Trait scales

We calculated the mean reliability rating for each trait scale (PC, NC, PE, NE) for each target (slow, average, fast) in the same way that we did for the pilot study. The scales all had high average reliability across all three target speeds, with average Cronbach's alphas of α_PC_ = .87(α_slow_ = .89, α_average_ = .89, α_fast_ = .84), α_NC_ = .84(α_slow_ = .84, α_average_ = .90, α_fast_ = .79), α_PE_ = .83(α_slow_ = .80, α_average_ = .89, α_fast_ = .80), and α_NE_ = .81(α_slow_ = .67, α_average_ = .85, α_fast_ = .75).

#### Positive vs. Negative Traits

**Figure 2:**
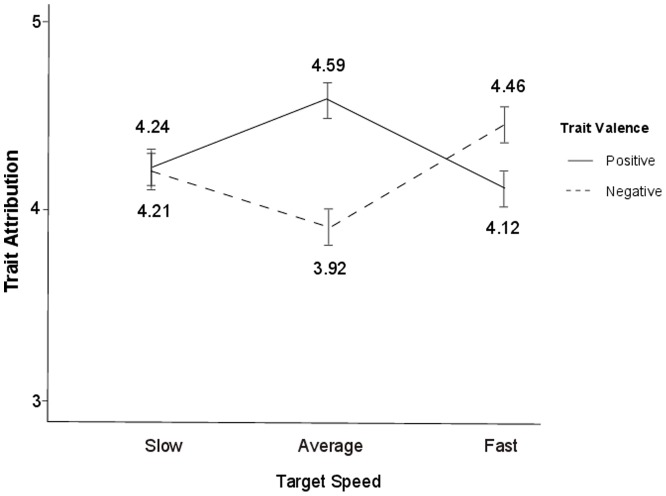
Attribution of targets' capacity for positive traits and negative traits, Main Study. Ratings were made from 1 (Not at all) to 7 (Extremely).

We first examined the differences between positive and negative traits, collapsed across cognitive/emotional dimensions. These means were analyzed by a 2 (Valence: Positive, Negative)×3 (Speed: Slow, Average, Fast) repeated measures ANOVA. There was a marginal effect of valence, *F* (1, 95) = 3.63, *p* = .06, η^2^_partial_ = .04, and there was no main effect of speed, *F* (2, 95) = 4.63, *p* = .63. The predicted interaction between valence and speed was significant, *F* (2, 95) = 21.31, *p*<.001, η^2^_partial_ = .18 (see Figure 2). We followed up this interaction with two separate quadratic contrasts for positive and negative traits on speed. The results of both contrasts were consistent with the timescale halo hypothesis. First, the quadratic contrast for positive traits was significant, *F*(1, 95) = 21.04, *p*<.001, η^2^_partial_ = .18, such that average-speed targets received higher ratings (*M* = 4.59, *SD* = .87) than the slow (*M* = 4.24, *SD* = .95) and fast targets (*M* = 4.12, *SD* = .89). Second, the quadratic contrast for negative traits was also significant, *F* (1, 95) = 18.36, *p*<.001, η^2^_partial_ = .16. As predicted, means for negative traits showed the opposite quadratic pattern than positive traits, where average speed targets received lower ratings (*M* = 3.92, *SD* = 1.07) than slow (*M* = 4.21, *SD* = .92) or fast targets (*M* = 4.46, *SD* = .91).

#### Cognitive vs. Emotional Traits

We also tested whether the same patterns would hold separately for the Cognitive and Emotional scales. We first examined the cognitive trait ratings with a 2 (Valence: Positive, Negative)×3 (Speed: Slow, Average, Fast) repeated measures ANOVA. These results revealed a significant main effect for both valence, *F*(1, 95) = 14.66, *p*<.001, η^2^_partial_ = .13, and speed, *F*(2, 95) = 5.52, *p*<.01, η^2^_partial_ = .06. However, these main effects were qualified by a significant interaction of valence and speed, *F*(2, 95) = 4.94, *p*<.05, η^2^_partial_ = .05. We followed this interaction with two separate quadratic contrasts on positive and negative traits. Positive-cognitive traits displayed the predicted inverse-U pattern, *F*(1, 95) = 4.53, *p*<.05, η^2^_partial_ = .05, with lower attributions for slow and fast targets relative to average-speed targets. Also as predicted, negative-cognitive traits displayed the U-shape pattern, *F*(1, 95) = 4.55, *p*<.05, η^2^_partial_ = .05, with higher attributions to slow and fast targets relative to average-speed targets (see Table 2 for means of individual cognitive traits).

**Table 2:** Perceived capacity for traits by target speed, Main Study.

			Target Speed					
			Slow		Average		Fast	
Trait	Valence	Dimension	Mean	(SD)	Mean	(SD)	Mean	(SD)
*Clever*	Positive	Cognitive	4.60	(1.30)	4.54	(1.08)	4.53	(1.06)
*Creative*	Positive	Cognitive	4.15	(1.28)	4.42	(1.12)	4.28	(1.19)
*Imaginative*	Positive	Cognitive	4.25	(1.37)	4.34	(1.13)	4.21	(1.13)
*Smart*	Positive	Cognitive	4.30	(1.15)	4.75	(1.01)	4.75	(1.10)
*Compassionate*	Positive	Emotional	3.96	(1.22)	4.57	(1.08)	4.24	(1.11)
*Helpful*	Positive	Emotional	3.84	(1.34)	4.76	(1.04)	3.76	(1.37)
*Loyal*	Positive	Emotional	4.27	(1.19)	4.66	(0.96)	4.17	(1.03)
*Patient*	Positive	Emotional	5.07	(1.57)	4.69	(1.15)	3.02	(1.60)
*Critical*	Negative	Cognitive	3.97	(1.46)	4.05	(1.27)	4.67	(1.31)
*Deceptive*	Negative	Cognitive	4.08	(1.37)	4.01	(1.33)	4.24	(1.25)
*Fault-finding*	Negative	Cognitive	3.95	(1.44)	3.93	(1.26)	4.56	(1.29)
*Scheming*	Negative	Cognitive	4.11	(1.37)	3.92	(1.39)	4.47	(1.24)
*Irritable*	Negative	Emotional	4.18	(1.43)	3.75	(1.37)	4.81	(1.40)
*Jealous*	Negative	Emotional	3.92	(1.23)	3.92	(1.14)	4.20	(1.20)
*Lonely*	Negative	Emotional	5.31	(1.26)	4.00	(1.44)	4.27	(1.37)
*Nervous*	Negative	Emotional	4.91	(1.40)	3.84	(1.42)	4.17	(1.51)

*Means and standard deviations of the ratings given for each target assessing their capacity for various mental traits at different walking speeds.*

For the emotional trait attributions, a 2 (Valence)×3 (Speed) repeated measures ANOVA revealed the same pattern of results. We found no main effect of valence, *F*(1, 95) = .10, *p* = .76, η^2^_partial_ = .001, or speed *F*(2, 95) = 2.44, *p* = .09, η^2^_partial_ = .03. More importantly, there was an interaction between valence and speed, *F*(2, 95) = 32.55, *p*<.001, η^2^_partial_ = .26 (see Table 2 for means of individual emotional traits). We conducted separate quadratic contrasts for the positive and negative emotional traits. Positive-emotional traits displayed the predicted inverse-U pattern, *F*(1, 95) = 32.57, *p*<.01, η^2^_partial_ = .26, such that average-speed targets were rated more highly on positive-emotional traits than slow and fast targets. Negative-emotional traits displayed the predicted U-shape pattern, *F*(1, 95) = 28.86, *p*<.01, η^2^_partial_ = .23, such that slow and fast targets elicited stronger negative attributions than average-speed targets.

#### Mediation analysis

The results of Study2 replicate those of the pilot study, consistent with the timescale halo predictions. Next, we conducted mediation analyses as a more direct test of the timescale halo hypothesis, using the global evaluations of each target. We used a multi-level mediation approach to account for the repeated-measure nature of the data [31]. Mixed model mediation assumes a linear relation between the model's variables, but here we expect a non-linear relation between a target's perceived-speed (the independent variable) and overall target evaluations (the mediating variable) as well as specific trait attributions (the dependent variable). We therefore re-coded speed ratings as the absolute deviations from the scale midpoint, following the recoding performed in the original timescale bias paper [5]. The independent variable in our mediation model may therefore be understood as the relative abnormality of target-speed, with larger values reflecting greater deviation from average-speed.

Because the primary hypothesis predicts opposite effects of speed for positive vs. negative traits, we conducted separate mediation for positive/negative traits. In both mediation analyses, we used subjective ratings of speed (deviation from normal speed) as the independent variable, global positivity towards the target as the mediating variable and specific trait attributions (positive or negative) as the dependent variable. As predicted, mediation analysis revealed a significant indirect effect of perceived speed abnormality on positive trait attributions through overall target evaluations, *indirect effect* = −.07, *SE* = .03, *p*<.05, accounting for 22.06% of the relation between speed abnormality and positive trait attributions. Similarly, we found a significant indirect effect of perceived speed abnormality on negative trait attributions through overall target evaluations, *indirect effect* = .11, *SE* = .05, *p*<.05, accounting for 63.20% of the relation between speed abnormality and negative trait attributions. Perceived deviation from normal speed predicted general negativity toward a target, which in turn predicted attributions for specific positive and negative mental characteristics.

### Discussion

In our primary study, we found that average-speed targets were rated higher on positive mental traits and lower on negative mental traits than either slow or fast targets. These patterns were found across both cognitive and emotional dimensions of mind. Moreover, these results provide support for the timescale halo hypothesis by demonstrating that the effect is mediated by global positivity toward each target. We also used observers' subjective judgments of relative target speed as the independent variable, rather than the absolute speed of the target. Greater perceived deviation from normal speed predicted more negative evaluations of the target in general, which thereby impacted judgments on the specific positive and negative mental traits. Thus, these results provide support for a timescale halo explanation by demonstrating that average speeds do not increase all perceptions of mental capacity, but rather lead to more positive global evaluations of a target.

## General Discussion

We revisited the timescale bias [5] to test whether the relation between target speed and attributions of mind is the result of a general halo effect. The present research found an interaction between speed and valence on judgments of mind: targets who moved at an average speed were perceived to have more positive characteristics of mind (e.g., clever, compassionate) and fewer negative characteristics of mind (e.g. deceptive, irritable) compared to slow or fast targets. Importantly, these effects were mediated by global evaluations of the target. Rather than seeming to have more mind in general, average-speed targets were evaluated more positively in general, which impacted attributions on a variety of specific mental characteristics. Together, these results provide evidence for a *timescale halo*.

### The Relation of the Timescale Halo to the Timescale Bias

The original research on the timescale bias observed a curvilinear pattern on attributions of “mind”, but results were limited to using positive-cognitive traits, such as “intentionality” and “intelligence.” We replicated this effect for positive-cognitive traits, but also expanded on the original research in two important ways. First, we demonstrated that the effect of movement speed on target attributions extends beyond cognitive attributes to emotional dimensions of mind. More importantly, we found the opposite curvilinear pattern for negative mental traits, evidence for a timescale halo effect. Thus, the present work revealed that deviations from normal movement speed elicit less favorable evaluations, which then influence judgments of specific mental traits, rather than relative movement speed directly affecting general judgments of mind.

It is also important to note that, in our studies, participants only rated human targets. In the original timescale studies, the targets included non-human such as animals, animations, or figurines. Therefore, our current “timescale halo” explanation might only apply to interpretations of the timescale bias concerning humans. The original Morewedge et al. studies also found that people attribute more mental capabilities to dogs and cats (which move at speeds similar to humans) more than turtles or hummingbirds (which move at speeds different than humans). The present research did not. The present research did not examine non-human targets, so we cannot say for certain whether our findings extend to those situations. Future research could replicate our studies using non-human targets to examine the generalizability of the timescale halo. However, if the timescale halo only applies to situations involving human targets, it is nonetheless, an important condition to know as humans frequently make judgments about other humans.

## Conclusion

We investigated whether the timescale bias (increased attribution of mind to average speed humans) was the result of a more general halo effect. Rather than a special perception of mind in average-speed targets, the timescale bias appears to vary as a function of the general positivity felt toward average-speed targets, compared to slow or fast moving targets. People walking at average speeds are more likely to receive positive attributions and less likely to elicit negative trait attributions. In other words, mental attributions vary by target speed in a *timescale halo*.
